# Late Presentation of Uterine Rupture: A Case Report

**DOI:** 10.7759/cureus.5950

**Published:** 2019-10-21

**Authors:** Meher B Ali, Mashal B Ali

**Affiliations:** 1 Gynecology and Obstetrics, Dow University of Health Sciences, Karachi, PAK

**Keywords:** peritonitis, uterine rupture, vaginal birth after cesarean

## Abstract

Uterine rupture is a rare disease, which usually presents in the form of a life-threatening emergency. It occurs most commonly after a vaginal birth after cesarean (VBAC) and is characterized by complete separation of the uterine incision throughout most of its length, involving all layers of the uterus. We present the case of uterine rupture in which the patient had no acute signs of rupture; instead, she presented with symptoms of generalized peritonitis. She presented 23 days after VBAC, with complaints of slight vaginal bleeding and a palpable abdominal mass. Leukocyte and platelet counts were raised, suggesting infection, which occurred due to the spread of fluid from the uterus to the abdomen through the scar defect. A total abdominal hysterectomy was performed due to widespread adhesions and a non-viable uterus. Our case was unusual as the presentation was delayed, with no acute symptoms of either uterine rupture or peritonitis. Uterine rupture can be fatal if not recognized and managed promptly.

## Introduction

Uterine rupture is a rare and often fatal complication of prior cesarean section (C-section), occurring in 0.3% of such cases. It is characterized by the complete separation of the uterine incision throughout most of its length, involving all layers of the uterus. The risk increases drastically following vaginal birth after cesarean (VBAC), and it occurs in 0.47% of cases of VBACs [1]. Classical C-section, now rarely performed, is associated with greater chances of rupture than a lower segment C-section ( (LSCS) [2]. In an unscarred uterus, the cause is mainly traumatic or iatrogenic. Its incidence in primigravida is so infrequent that an undisclosed pregnancy or previous uterine surgery needs to be probed in such cases [2]. Uterine rupture usually presents acutely during labor, or right after delivery, with fetal bradycardia, slowing of contractions, abdominal pain, vaginal bleeding, maternal tachycardia, hypotension, and/or uterine atony [1]. We present the case of a uterine rupture occurring after VBAC in which there were no acute signs of rupture and, instead, the patient presented with mild symptoms of generalized peritonitis. 

## Case presentation

A 30-year-old patient of parity three was admitted with complaints of mild vaginal bleeding and a palpable abdominal mass for 23 days, which had developed shortly after a VBAC. The patient’s second child had been delivered via C-section two years ago due to breech presentation, after which a third, healthy baby had been delivered 23 days ago through spontaneous vaginal delivery (SVD) by a midwife at home. There were no complaints of pain, fever, or any other symptoms. Vitals were stable. On abdominal examination, fundal height was found to be 32 weeks, and a soft large mobile mass in the right adnexal region was found whose lower limit was not reachable. There was mild bleeding during a vaginal examination. Blood tests yielded decreased values of hemoglobin [9.9 g/dL; normal range (N): 12-16 g/dL]; a mean corpuscular volume of 74.5 fL/cell (N: 80-96 fL/cell); mean corpuscular hemoglobin of 24.5 pg/cell (N: 27-33 pg/cell); mean corpuscular hemoglobin concentration of 32.9 g/dL (N: 33-36 g/dL), and activated partial thromboplastin time (aPTT) of 21.1 seconds (N: 60-70 seconds). Lab results showed elevated total leukocyte count (TLC) and platelet count (PC) with values of 15.8×10^9 ^cells/L (N: 4.3-10.8×10^9^ cells/L) and 700×10^9 ^cells/L (N: 150-400×10^9^ cells/L), respectively. An ultrasound of the abdomen and pelvis showed a separated lower uterine segment stitch line and collection of fluid in the abdominal pockets and rectouterine pouch. The estimated fluid level was 1.5-2 liters. An exploratory laparotomy was performed by the surgical unit team via a midline incision, during which the whole gut including the bladder, ovaries, and uterus was found to be plastered with thickened adhesions. The patient was diagnosed with puerperal sepsis and peritonitis, which was attributed to uterine rupture. Subsequently, 1.5-2 liters of straw-colored fluid was drained from the peritoneal cavity. Uterus was found to be coated with fibrinous pus flakes. A hematoma was present in the right adnexal region and the cesarean scar was dehiscent throughout its entire extent. A total abdominal hysterectomy (TAH) was then performed. After vault closure and securing of hemostasis, two drains were placed in the abdominal cavity for postoperative drainage. Following surgery, the patient was monitored and put on intravenous (IV) piperacillin-tazobactam, IV metronidazole, and IV tranexamic acid. These were continued for four days postoperatively. On the first postoperative day, TLC and PC decreased to 13.1×10^9 ^cells/L and 642×10^9 ^cells/L, respectively. Both drains were emptied, drawing 680 milliliters of fluid from the left drain and 395 milliliters of fluid from the right drain. The patient continued to be monitored for seven days during which her lab values became normal and she became clinically stable. The recovery period was uneventful.

## Discussion

Uterine rupture needs to be managed promptly due to its fatal complications. A delay in seeking medical attention can be costly because of the many threats VBAC poses to the life of the patient. Our patient presented to us 23 days after VBAC which she had undergone at home with the help of a local midwife. It is recommended that VBAC patients should have an adequately equipped staff for safe delivery with continuous fetal monitoring and facilities of emergency C-section and blood transfusion, in case of complications like uterine rupture [3].

**Figure 1:**
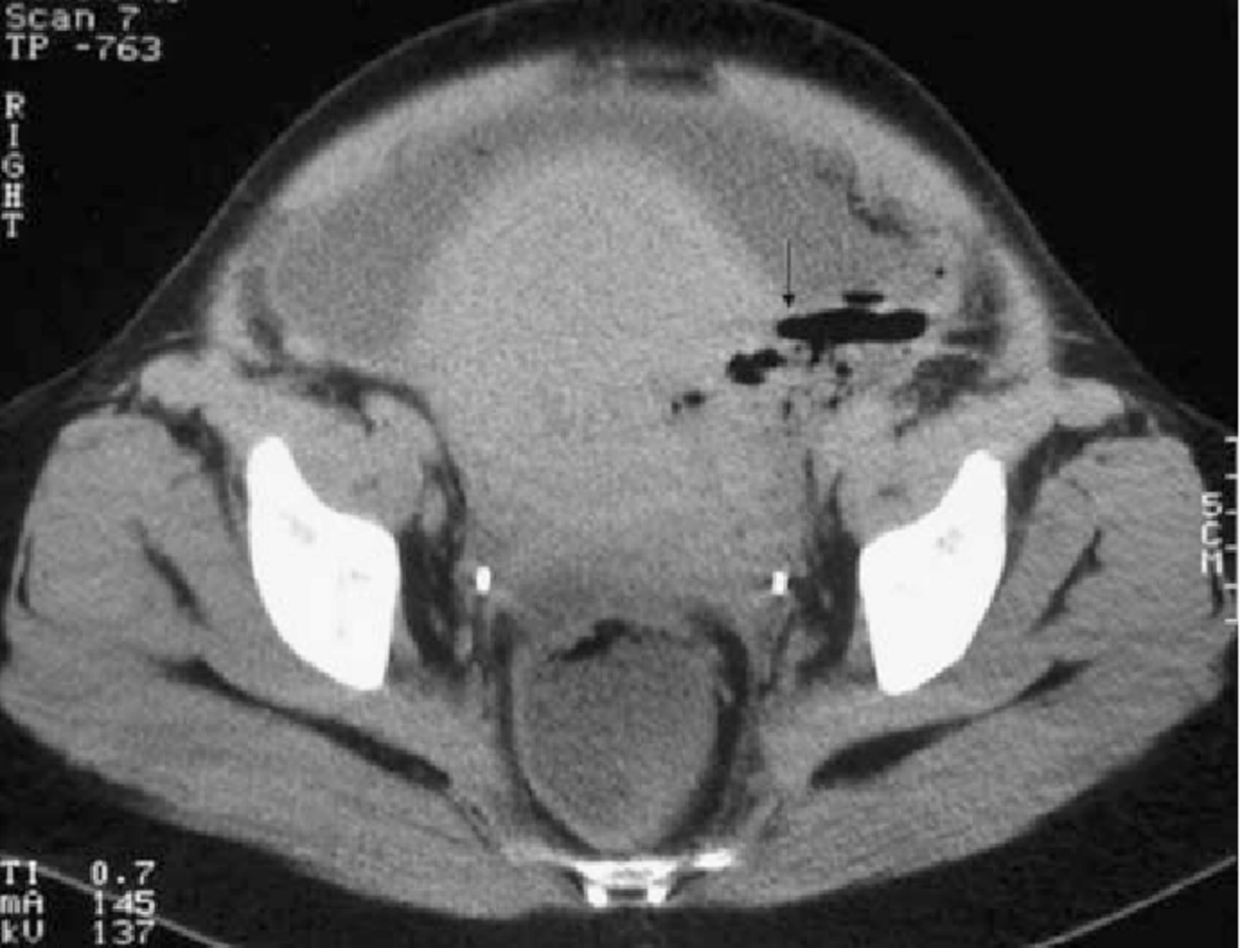
CT showing air bubbles spreading through the ruptured uterus into the abdominal cavity

The uterus descends into the pelvis after delivery at a rate of one cm per day and should be nonpalpable by two weeks postpartum. Thus, a fundal height of 32 weeks three weeks that was observed in our patient was considered an alarming sign that required immediate attention. Though uterine rupture usually manifests with its own acute signs and symptoms along with hemodynamic instability, our case informs us that it can present through mild symptoms of life-threatening complications like infections too. Infection of the female reproductive tract occurring from the end of labor until six weeks postpartum is defined as puerperal sepsis. In the presence of uterine rupture, the infection extends into the peritoneal cavity through the scar defect, leading to peritonitis. The patient can first present with vaginal bleeding, and then, on the involvement of the peritoneal cavity, symptoms of abdominal infection like abdominal pain, distension, or fever can occur. Peritonitis is a medical emergency as delay in diagnosis can lead to bacteremia and septic shock [4]. Our patient did not have any abdominal pain or fever on presentation. Generalized peritonitis was observed, which carries little to no classic symptoms of localized peritonitis. Lab reports showed an increased TLC and PC. A constantly raised TLC after delivery should lead to the differential diagnoses of a potential underlying infection, even if classic symptoms are not present. Similarly, a PC of greater than 500×10^9 ^cells/L is usually an indicator of an underlying inflammatory reaction, i.e., secondary thrombocytosis [5]. The first-line modality of investigating uterine rupture is an ultrasound scan [6]. Though its yield is low, it may indicate rupture like in our case, by showing the presence of fluid outside the uterus. CT and MRI are more accurate as they clearly show the dehiscent line (Figure 1 ) [6]. However, they are expensive and thus are not done routinely in low socioeconomic setups.

When a rupture is suspected on ultrasound by the presence of fluid in the pouch of Douglas and a dent in the uterus, it is advisable to go for an exploratory laparotomy directly [7]. In our case, an exploratory laparotomy yielded widespread adhesions around organs and showed the scar to be heavily infected and irreparable. This made conservative treatment impossible. Hence, a TAH was carried out after peritoneal lavage. The risk of uterine rupture necessitating a hysterectomy ranges from 14 to 33% [1]. A uterus-saving procedure is done only in patients whose uterus is viable and only when deemed not a threat to the life of the patient. For these patients, antibiotics, surgical repair, and debridement of necrotic tissue are the mainstay. Thus, it is necessary to set the treatment plan based on the conditions of each patient.

The frequency of C-sections nowadays means that most obstetricians will be encountering VBACs on a regular basis. There is still debate over the preferable mode of delivery after a prior C-section. Some studies have demonstrated that another C-section carries greater risk compared to VBAC. That said, there has been a decline in the number of women opting for VBACs [8].

## Conclusions

Even though uterine rupture is rare, its accompanying complications like peritonitis can be fatal if not recognized and managed promptly. Our case was unusual because the presentation was delayed, with no acute symptoms of either the uterine rupture or peritonitis. It is necessary to consider the risk of rupture when attempting VBAC and to follow up on the patient to prevent complications, even when the symptoms indicative of either rupture or infection are observed to be mild.

## References

[1] Guise JM, Eden K, Emeis C (2010). Vaginal birth after cesarean: new insights. Evid Rep Technol Assess (Full Rep).

[2] Turner MJ (2002). Uterine rupture. Best Pract Res Clin Obstet Gynaecol.

[3] Royal College of Obstetricians and Gynaecologists (2019). Royal College of Obstetricians and Gynaecologists: Birth after previous caesarean birth (Green-top Guideline No. 45). https://www.rcog.org.uk/en/guidelines-research-services/guidelines/gtg45/.

[4] Al Qahtani N, Al Taifi H (2017). Caesarean section scar dehiscence with peritonitis: Does late surgical intervention minimize the risk of hysterectomy? CS dehiscence with peritonitis. Int J clinical & case.

[5] Griesshammer M, Bangerter M, Sauer T, Wennauer R, Bergmann L, Heimpel H (1999). Aetiology and clinical significance of thrombocytosis: analysis of 732 patients with an elevated platelet count. J Intern Med.

[6] Has R, Topuz S, Kalelioglu I, Tagrikulu D (2008). Imaging features of postpartum uterine rupture: a case report. Abdom Imaging.

[7] Dhar RS, Misra R (2012). Postpartum uterine wound dehiscence leading to secondary PPH: unusual sequelae. Case Rep Obstet Gynecol.

[8] Madaan M, Agrawal S, Nigam A, Aggarwal R, Trivedi SS (2011). Trial of labour after previous caesarean section: the predictive factors affecting outcome. J Obstet Gynaecol.

